# Improving power ramp rate of a coal-fired power plant by a bypass steam accumulator

**DOI:** 10.1016/j.heliyon.2024.e32412

**Published:** 2024-06-04

**Authors:** Hongyu Ding, Sibian Ding, Qingxiong Tan, Cheng Zhang, Qingyan Fang, Tao Yang

**Affiliations:** a School of Energy and Power Enginnering, Huazhong university of science and technology, 430074 Wuhan, China; b State Key Laboratory of Coal Combustion, Huazhong university of science and technology, 430074 Wuhan, China

**Keywords:** Deep peak-shaving, Flexible generation, steam accumulator, Power ramp rate

## Abstract

The increasing penetration of high-volatility renewable energy sources in the power system presents higher demands for flexibility from coal-fired power plant (CFPP). To enhance the flexibility of CFPPs, researchers have conducted a significant amount of thermal-system-level research in recent years on increasing system peak shaving depth. However, the load ramp rate of CFPPs under deep peak shaving is rarely discussed, despite its significance to the overall flexibility performance of CFPPs. This paper proposes a steam accumulator storage system integrating to the turbine's bypass system. The steam accumulator charges directly with working fluid from the live steam or reheat systems and discharge to the turbine, responding quickly to power ramp commands. A steady state model and a dynamic model of the proposed system were built and validated, and the calculation shows that the proposed scheme provides a load change of +2.13 % Pe and −8.3%Pe during a round-trip with a power efficiency of 63.6 % at a unit load of 40 % THA. The unit's load increase rate under coordinated control was enhanced by 1.5 % Pe/min, reaching 3 % Pe/min, using the proposed steam accumulator without revising the original controls, and the load decrease rate reached at least 5 % Pe/min. The results indicate that the proposed system provides a straightforward, easy-to-implement, and efficient solution for enhancing the load ramp rate of CFPPs at low loads.

## H I G H L I G H T S

• A novel steam accumulator is integrated to the bypass system of the power plant.
• A dynamic supercritical coal-fired power plant model is developed based on the reference plant for the simulation.
• The load increase rate is enhanced by 1.5 % Pe/min at 40 % THA condition.
• The load decrease rate reached at least 5 % Pe/min at 40 % THA condition.

## 1 Introduction

The market penetration of renewable energy is increasing rapidly. In the first three quarters of 2022, China's installed capacity of renewable energy increased by 90.36 million kilowatts, accounting for 78.8 % of the country's new installed power generation capacity [1]. Global renewable energy generation could surpass coal by 2024 [2]. The rapid growth of renewable energy in the power system has led to an increasing demand for stable power sources and power flexibility in the grid. Flexible capacity in the power grid compensate for the unreliability of renewable energy sources by quickly balancing power generation and demand [3], thereby allowing a higher share of renewable energy in the electricity system. The CFPP's flexibility is crucial in power systems, particularly in countries with abundant coal resources, such as China [4], Poland [5], India [6].

The flexibility of a CFPP can be rated by start-up times, ramping rate, and peak shaving capacity [7]. Conventional methods to increase plant flexibility includes utilizing the thermal heat within the combustion-flue gas, metal, and water-steam system of the plant. Gao et al. [8] designed a coordinated control system control (CCS) strategy named advanced energy balance. The strategy utilizes the energy stored in fuel and drum of a circulating fluidized bed boiler, successfully improved the control quality of unit load. Wang et al. [9] modified the water-fuel ratio control strategy of a supercritical power plant to activate the thermal energy stored in boiler metal, successfully improving the flexibility of the unit. Zhao et al. [10] studied the utilization of stored heat in regenerative systems, and found that high pressure extraction steam throttling led to the greatest power ramp rate of 6.31 % Pe/min. In contrast, low pressure extraction steam throttling yielded a power ramp rate of 1.88 % Pe/min, condensate flow reduction result in a power ramp rate of 1.26 % Pe/min. However, due to the temperature stress in the regenerative heaters caused by high pressure extraction steam throttling, the latter two means are much more preferable [11]. Above conclusions are made all under the designed load level of researched units.

The recent acceleration of renewable energy development has frequently required CFPPs to implement deep peak-shaving operations, which significantly reduces plant flexibility [12]. Shi et al. [13] reported that power ramp capacity provided by high pressure extraction steam throttling under unit load of 40%THA was reduced to 1.4%Pe compared to 7.33%Pe, and low pressure extraction steam throttling was reduced to 0.6 % Pe compared to 1.93 % Pe at 100 % THA [10], mainly because of low TES in the boiler and regenerative system, as the thermal inertia of a CFPP limited the flexibility provided by adjusting the feed rate of coal.

Integrating external TES is an interesting approach to strengthen the plant's overall flexibility. This is because thermal storage can bypass the processes of milling, combustion, and heat exchange. Moreover, the deterioration of combustion conditions at low loads will not affect the operation of Thermal Energy Storage (TES) systems. The most popular TES technology in power generation should be indirect TES such as two-tank molten salt [[14], [15], [16]]. In a work cycle of indirect TES, heat energy in the thermal system is captured by heat exchangers, thus stored in working medium of a TES. The stored heat energy is then returned to the thermal system when needed. The round-trip efficiency (RTE) is defined by the heat loss during heat exchange and storing process in the prospective of energy.

Li et al. [17] summarized two general methods to integrate heat energy from TES to CFPP: heating steam to directly power up the turbine, and heating water to substitute extraction steam. Cao et al. [18] proposed an additional thermodynamic cycle, improving load flexibility by a separate turbine powered by a TES. Krüger et al. [19] found that molten salt and solid medium TES can reduce the minimum load of a CFPP by 3–4%Pe, while the RTE ranges between 46 and 82 %. Above studies demonstrated the feasibility of integrating TES technologies in CFPPs to improve plant flexibility. However, these studies consider the average performance of energy storage over the course of several hours of charging and discharging. Due to the high thermal inertia of indirect thermal storage technologies such as molten salt and phase change storage, their flexibility isn't readily accessible.

Due to the thermal inertia of the heat exchangers, the response time of molten salt TES is usually at a range of 10 min [20]. Judging from the thermal conductivity of the working medium, this latency is even longer for latent heat storage [21] and concrete heat storage [22]. Metal latent heat storage has high thermal conductivity and low heat transfer inertia but is too expensive for power generation [23]. For these reasons, CFPPs with indirect TES technologies can provide better long-time peak-shaving services, but their ability to provide primary frequency reserve is not improved as much. Apart from long response delay, extra heat exchangers are required when introducing indirect TES technologies into CFPPs. This makes smaller indirect TES less cost efficient. Çam et al. [24] conducted a study on the participation of CFPPs with TES in the German electricity market, and found that considerable profits can be achieved by integrating 0.5–1 h of TES storage time, while the profits maximize at a storage time of 2∼3 h. Yin et al. [25] estimated an optimal integrated battery volume of 3 % Pe for CFPPs in Guangdong province to provide primary frequency reserve. Direct TES such as steam accumulator (SA) is a cost-effective approach to provide fast energy output [26]. Richter et al. [27] proposed to introduce a SA storing extracted superheated/reheated steam, and release saturated steam to heat the feed water substituting high pressure extraction steam. The design uses a SA with a volume of 1331 m^3^, providing 30 min of heat storage. Stefanović et al. [28] proposed a similar system using SA to substitute extraction steam in low pressure regenerative heaters, with a accumulator volume of 600 m^3^.

The literature review indicates that the CFPPs encounter difficulties on load flexibility under low load conditions, because of low heat storage in the system at low loads. Some studies proposed using external TES, for instance, molten salt storage, latent heat storage and direct steam storage, to enhance plant flexibility. Indirect TES technologies allow large storage capacity. However, the high thermal inertia of the indirect TES makes it less suitable for primary frequency reserve or boosting power ramp rate during transient operations. They also require expensive extra heat exchangers. Direct TES technologies, such as SA, have quick response time and low investment demand. Thus, they are suitable for improving plant flexibility during transient operations. However, the existing studies on integrating SAs into CFPP focus on substituting extraction steam in the regenerative systems. This method is similar to the extraction steam throttling regulation or feed water bypass regulation. Therefore, it should provide similar flexibility reserve. However, the mass flow rate of extraction steam is severely reduced at low loads, which worsens the performance of the integrated SA. Besides, substituting extraction steam with the SA precludes the ability to regulate either the extraction steam throttling or the feed water bypass.

This paper presents a novel solution for improving the power ramp ability of a CFPP. The proposed system incorporates a SA storage system integrated with the plant's bypass steam system. It charges from the live steam pipes and releases saturate steam to the cold reheat steam (CRH) pipes. The charge-discharge cycle is located in bypass system, hence the name “bypass steam accumulator”. Compared with the substituting extraction steam scheme, the bypass steam accumulator integration scheme allows for a greater mass discharge rate, thereby providing a significant degree of flexibility to enhance the plant's overall performance at low loads. To further analyze the system performance, simulations are made based on data from a reference CFPP, including a steady state model to analyze its theoretical performance, as well as a transient model to test its performance during transient process. The result and analysis are made under a unit load of 40%THA, which is a typical load level under deep peak-shaving.

**Table:** Nomenclature^a^

*Abbreviations*								*ε*	expansion ratio, 1
					*ϵ*_turbine_				turbine exergy efficiency, %
*Mathematical symbols*									
CCS		coordinated control system	*A*				Area per mass flow rate, m^2^·s/kg	*η*	efficiency, %
CFPP		coal-fired power plant	*c*				velocity, m/s	*μ*	velocity loss factor, 1
CRH		cold reheat steam	*E*				latent heat volume density, kJ/(kg·m³)	*π*	compression ratio, 1
DSA		distributed control system	H(·)				specific enthalpy function, J/kg	*β*	max liquid volume ration, 1
H		regenerative heater	*H*				enthalpy, J	*Subscripts*	
HD		deaerator	*h*				specific enthalpy, J/kg	1	begin
HRH		hot reheat steam	*m*				mass flow rate, kg/s	2	end
HPT		high-pressure turbine	P(⋅)				pressure function, Pa	d	diffuser
IPT		intermediate-pressure turbine	*p*				pressure, Pa	is	isentropic
LPT		low-pressure turbine	S(⋅)				specific entropy function, kJ/(kg·K)	mi	motive flow inlet
RTE		round-trip efficiency	*s*				specific entropy, kJ/(kg·K)	mix	mix output
SA		steam accumulator	*t*				time, s	sat	saturation
SG		shaft seal heater	*Greek symbols*					sb	secondary back
SC		steam cooler	*v*				specific volume, m^3^/kg	si	secondary inlet
TES		thermal energy storage	*w*				entrainment ratio, 1	tot	total
THA		turbine heat acceptance							

a The formatting here does not show up correctly in the preview, and it is difficult using online editor to make complex manipulations of the table.

## 2 Integration of the steam accumulator

### 2.1 System description

**Fig. 1:**
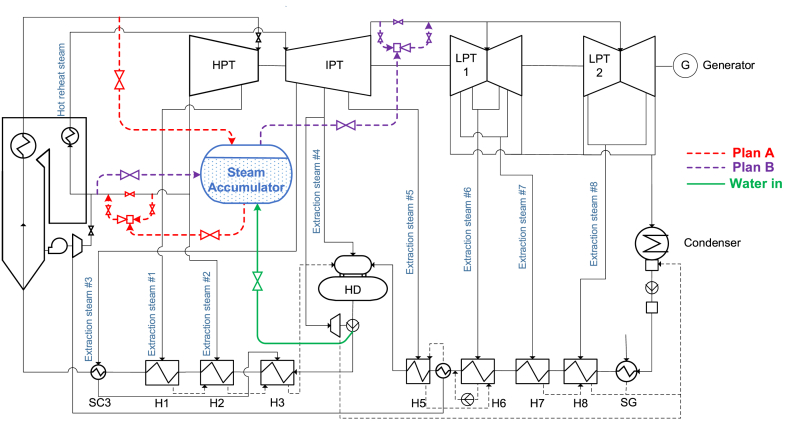
Scheme of the proposed bypass SA integrate with the reference CFPP.

A 660 MW supercritical CFPP in Zhoukou, Henan Province is selected as reference case (see Fig. 1). It has a single-reheat boiler, with three high pressure regenerative heaters (H1–H3), four low pressure regenerative heaters (H5–H8), and one deaerator (HD). A steam cooler (SC3) is applied to H3, and a shaft seal leakage steam heater (SG) is installed before H8, to increase energy efficiency. There are two induced draft fans driven by steam turbines which are powered by CRH. The steam used is then sent to a heat exchanger between H5 and H6, and finally H5.

When below 50%THA, only one induced draft fan is put into action. Also, two steam turbines are independently powered by extraction steam from intermediate pressure turbine (IPT) to drive two feed water pumps. The steam used is finally sent to condenser hot well. When below 50%THA, only one steam powered water pump is put into action. And when below 20%THA, both feed water pumps cease operation, and are replaced by an electric feed water pump. The reference CFPP adopts a P shaped once-through boiler, which turns into wet operation when below 30 % THA.

### 2.2 Designing the charge/discharge process

**Fig. 2:**
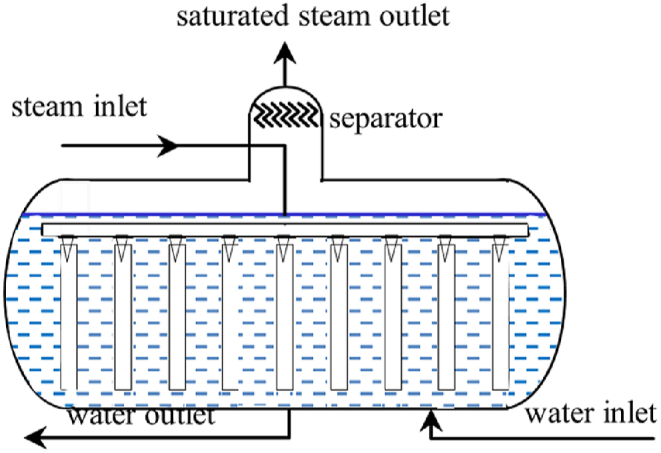
The structure of a typical SA.

The Fig. 2 shows the structure of a typical SA. During a charge-discharge working cycle, the SA is first filled with water via the feed water pump, and then charged by a steam source, which can be live steam, reheated steam or steam extracted from the turbine. The pressure in the SA rises as the charged steam heats the water in the SA, resulting a higher saturating pressure. When the pressure inside the SA is sufficiently higher than the discharge outlet, the SA can be discharged by releasing saturated steam, leading to a pressure fall, then the water evaporates to steam. The discharge continues until the SA pressure approaches the discharge outlet, preventing further evaporation.

Due to the reduced extraction steam mass flow rate under low load, little flexibility can be achieved by substituting extraction steam with SA. To further improve the power ramp rate of the plant, a better solution should be redirecting the released steam to the live steam or reheat steam pipes.

Using the extraction steam as the steam source could be an efficient choice, but without modifications to the extraction outlet of the turbines, the charge rate will be limited, and measures such as feed water bypass must be taken to protect the regenerative heaters as most of the extraction steam is redirected to the SA. Considering that the discharging of the SA is driven by pressure difference, the discharge outlet pressure must be lower than the steam source.

When the unit load is below 40 %, it is typically considered to be in a deep peak phase. At this load level, the unit's power ramp capability is severely degraded, and therefore requires additional flexibility sources. Taking all these considerations into account, the possible SA integration system scheme is shown in Table 1.

**Table 1:** Possible integration schemes.

Parameter	Scheme A	Scheme B
Steam source of charging process	Live steam	CRH
Steam source pressure at 40%THA	12.5 MPa	2.43 MPa
Discharge outlet	CRH	LPTs
Discharge outlet pressure at 40%THA	2.43 MPa	0.191 MPa

### 2.3 Modelling

To obtain reasonable design parameters and make a preliminary assessment of storage performance, simplified steady-state models of the SA and the thermocompressor are developed:

The SA is described by equations (1), (2), (3), (4). The absorption and generation of steam depends on the heat transfer between water and steam, which is related to the inner structure of SA. In equation (1), the heat transfer between water and vapor is assumed to be fast enough, that the water and vapor inside the SA have the same temperature at container pressure *p*. The assumption is accurate enough for engineering simulations, but temperature difference exists in real SAs. If the SA discharge or charge too fast, this assumption may cause distortion in the transient [29].

(1) $$\begin{array}{c} t_{\text{steam}}=t_{\text{water}}=t_{\text{sat}} \end{array}$$

In equation (2), the conservation of enthalpy in the SA writes:

(2) $$\begin{array}{c} \frac{dH_{\text{tot}}}{dt}=\frac{dH_{\text{water}}}{dt}+\frac{dH_{\text{steam}}}{dt}+\frac{V_{\text{tot}}dp}{dt} \end{array}$$

In equation (3), the conservation of mass in the SA writes:

(3) $$\begin{array}{c} m_{\text{tot}}=m_{\text{water}}+m_{\text{steam}} \end{array}$$

In equation (4), the conservation of volume in the SA writes:

(4) $$\begin{array}{c} V_{\text{tot}}=V_{\text{water}}+V_{\text{steam}} \end{array}$$

**Fig. 3:**
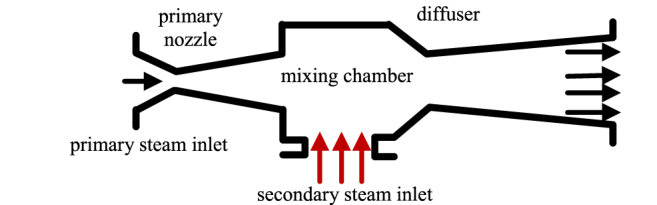
The structure of a thermocompressor.

Thermocompressors are implemented to the SA's outlet, mixing the steam provided by the SA and the turbine's steam mainstream. The thermocompressors are described by equations (5), (6), (7), (8), (9), (10), (11), (12), (13), (14), (15), (16), (17), (18), (19), (20), (21), (22), and Fig. 3 shows the typical structral of a thermocompressor [30]. Where *m* stands for mass flow rate, index “mi” stands for motive flow at the inlet, index “si” stands for secondary flow at the inlet. In the following equations, *p* stands for fluid pressure, *h* stands for specific enthalpy, *v* stands for specific volume, *s* stands for specific entropy, *c* stands for fluid velocity, and *η* stands for isentropic efficiency.

equations (5), (6), (7), (8), (9), (10), (11), (12), (13), (14), (15), (16), (17), (18), (19), (20), (21), (22) are based on the following assumptions.

• The thermocompressor is split to three parts: the primary nozzle, the mixing chamber, and the exit diffuser. Each part is considered a lumped system.
• Velocities at the primary and secondary inlet are neglected.
• In both inlets and the outlet, all losses such as friction, boundary layer separation and heat loss depend on isentropic efficiency.
• In the mixing chamber, all losses depend on friction, represented by velocity loss.

The entrainment ratio *w* is defined by equation (5)

(5) $$\begin{array}{c} w=\frac{m_{\text{si}}}{m_{\text{mi}}} \end{array}$$

Assume the motive flow experienced a pressure drop going through primary nozzle adiabatically, the index “is” stands for isentropic, the index “mb” stands for backend at primary nozzle zone, and function H(·) gives the enthalpy of the steam stream at certain fluid property:

(6) $$\begin{array}{c} p_{\text{mb}}=0.98p_{\text{mi}} \end{array}$$

(7) $$h_{\text{mb},\text{is}}=H\left(p_{b},s_{\text{mi}}\right)$$

(8) $$h_{\text{mb}}=h_{\text{mb},\text{is}}-\eta_{\text{mb}}\left(h_{\text{mi}}-h_{\text{mb},\text{is}}\right)$$

The steam velocity *c*_mb_ at the inlet:

(9) $$c_{\text{mb}}=\sqrt{2\left(h_{\text{mi}}-h_{\text{mb}}\right)}$$

Average flow area per total mass flow rate *A*_mb_ writes:

(10) $$A_{\text{mb}}=\frac{v_{\text{mb}}}{c_{\text{mb}}\left(1+w\right)}$$

Similarly, equations (11), (12), (13), (14) gives the secondary inlet backend (using the index “sb”) states:

(11) $$\begin{array}{c} h_{\text{sb},\text{is}}=H\left(p_{\text{mb}},s_{\text{si}}\right) \end{array}$$

(12) $$\begin{array}{c} h_{\text{sb}}=h_{\text{si}}-\eta_{\text{sb}}\left(h_{\text{si}}-h_{\text{sb},\text{is}}\right) \end{array}$$

(13) $$c_{\text{sb}}=\sqrt{2\left(h_{\text{si}}-h_{\text{sb}}\right)}$$

(14) $$\begin{array}{c} A_{\text{sb}}=\frac{w_{\text{sb}}}{c_{\text{sb}}\left(1+w\right)} \end{array}$$

It is assumed that the mixing of the primary and secondary streams occurs exclusively within the mixing chamber. In equations (15), (16), (17), (18), the index “mix” stands for the mixed steam stream, and the function S(⋅) gives the entropy of the steam at certain state.

The conservation of mass writes:

(15) $$\begin{array}{c} v_{\text{mix}}=\left(A_{\text{mb}}+A_{\text{sb}}\right)c_{\text{mix}} \end{array}$$

Considering speed loss *μ*, the conservation of momentum writes:

(16) $$c_{\text{mix}}=\mu \left(\left(p_{\text{mb}}-p_{\text{mix}}\right)\left(A_{\text{mb}}+A_{\text{sb}}\right)+\frac{c_{\text{sb}}w+c_{\text{mb}}}{1+w}\right)$$

The enthalpy equation writes:

(17) $$\begin{array}{c} h_{\text{mi}}+wh_{\text{si}}=\left(1+w\right)\left(h_{\text{mix}}+0.5c_{\text{mix}}^{2}\right) \end{array}$$

The entropy of the mix output can be found by property relation:

(18) $$\begin{array}{c} s_{\text{mix}}=S\left(p_{\text{mix}},h_{\text{mix}}\right) \end{array}$$

If the mixed steam is supersonic, additional equations should be added to take consideration of shock waves. But calculation shows that in the application of the proposed system, shock waves are not triggered. The mixed steam then expands in the diffuser and exits the thermocompressor. The index “d” is used to characterize the outlet steam of the diffuser.

The enthalpy equation writes:

(19) $$\begin{array}{c} h_{d}=\frac{h_{\text{mi}}+wh_{\text{si}}}{1+w} \end{array}$$

(20) $$\begin{array}{c} h_{d,\text{is}}=\eta \left(h_{d}-h_{\text{mix}}\right)+h_{\text{mix}} \end{array}$$

The outlet pressure *p*_d_ can be found by property relation P(⋅):

(21) $$\begin{array}{c} p_{d}=P\left(h_{d,\text{is}},s_{\text{mix}}\right) \end{array}$$

Finally, the compression ratio π can be defined by equation (22), this parameter is essential for the direct application of the SA to the turbine, because during the discharging process of the SA, the compression ratio raises the turbine's working pressure, which directly affects the power output of the unit:

(22) $$\begin{array}{c} \pi =\frac{p_{d}}{p_{\text{si}}} \end{array}$$

**Fig. 4:**
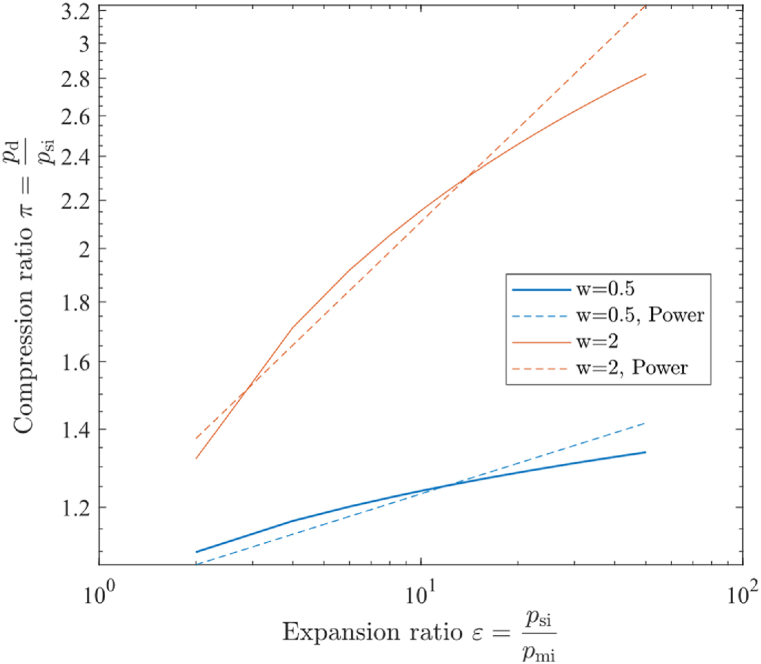
Expansion ratio - compression ratio correlation curve for the thermocompressor.

The above equations are calculated numerically, assuming all the isentropic efficiencies are η=0.9, and the velocity loss factor uses μ=0.98 as suggested in Ref. [30]. The pressure, enthalpy, and mass flow rate at both inlets are considered known input values, the equations give the pressure and enthalpy at the outlet as a result. equations (10), (14) gives the nominal geometric area of the thermocompressor, therefore equations (5), (6), (7), (8), (9), (10), (11), (12), (13), (14), (15), (16), (17), (18), (19), (20), (21), (22) adaptively provides reference designing parameters for the thermocompressor. The results are compared with the empirical correlation curve by Power [31] in Fig. 4, which demonstrates the correlation between expansion ratio $\varepsilon =p_{\text{si}}/p_{\text{mi}}$ and compression ratio π when responding to entrainment ratios *w* equal to 0.5 and 2. The maximum errors are 14.57 % for w=2 and 5.96 % for w=0.5. The results of the thermocompressor model are found to be in good agreement to the empirical correlation by Power under low expansion ratio, while discrepancies under high expansion ratio may be result from the constant speed loss at the mixing chamber.

## 3 Thermodynamic performance

The volume, the pressure-bearing capacity, and the speed of charging and discharging determines the performance of the integrated SA. Due to the SA's complex interactions with the water-steam cycle of the CFPP during operation transients, it is necessary to pre-determine these parameters of the SA from a thermodynamic perspective. The parameter design aims at a balanced RTE and ramping rate boost, while the discharge capacity is set to 30 min. The calculations are made at a unit load of 40 % THA using Ebsilon based on the following assumptions.

• The boiler runs at a fixed firing rate at 40 % THA, the live steam temperature and effective heat utilization of the boiler maintained at original values.
• The feed water rate remains the same, and additional water mass flow is added to or subtracted from condensate water to maintain the water level of the deaerator.
• The efficiency of the turbine is the same as at 40 % THA unit load.

The reheaters in the quasi-steady thermodynamic model are calibrated with the design parameters at 100 % THA provided by the reference plant.

The accuracy of the model is verified with temperature errors in regenerative systems as shown in Table 2. All errors are below 2 %, meaning the quasi-steady thermodynamic model is relatively accurate. Note that the results from SimStore model is considered accurate value as its thoroughly tested on the reference plant as mentioned later in section 4.

**Table 2:** Temperature errors in the regenerative system.

Node	SimStore model temperature (°C)	Ebsilon model temperature (°C)	Temperature error (%)
Economizer inlet	239	239.14	0.06
Heater #1 outlet	232	233.15	0.50
Heater #2 outlet	218.9	222.71	1.74
Heater #3 outlet	177.9	178.39	0.28
Deaerator outlet	142.8	144.1	0.91

The design pressure of the SA is determined by the steam parameters at the charging steam extraction point. One of the main exergy losses caused by the SA is the pressure drop of the charging steam, therefore it is better to have the design pressure close to or slightly higher than the charging steam. For this reason, the design pressure is set to 2.5 MPa when choosing CRH as charge steam source. However, manufacturing an accumulator vessel with a bearing pressure of 10 MPa or more is unlikely. The design pressure when charge with live steam is set at 8 MPa, as the live steam pressure drops to this fixed point below 30%THA.

During a round-trip, the SA takes in over heated steam, but gives out saturated steam. This means the steam charged will be less than the steam discharged. Therefore, a water refill must be done to restore the water level. Water refill can be done slowly to minimize the impact on the unit. However, in extreme cases, the SA may be required to operate frequently in a short period of time. Consider that in the worst case, the SA is required to complete a charge/discharge cycle in the shortest possible time. This requires the SA to be refilled, charged, and discharged at the maximum feasible rate. The water refill rate should meet the minimum flow rate of boiler feedwater, and the steam charge rate should meet the minimum flow rate of boiler reheater. Based on the boiler's operating regulations regarding steam bypass system, it is estimated to be 50 kg/s.

**Fig. 5:**
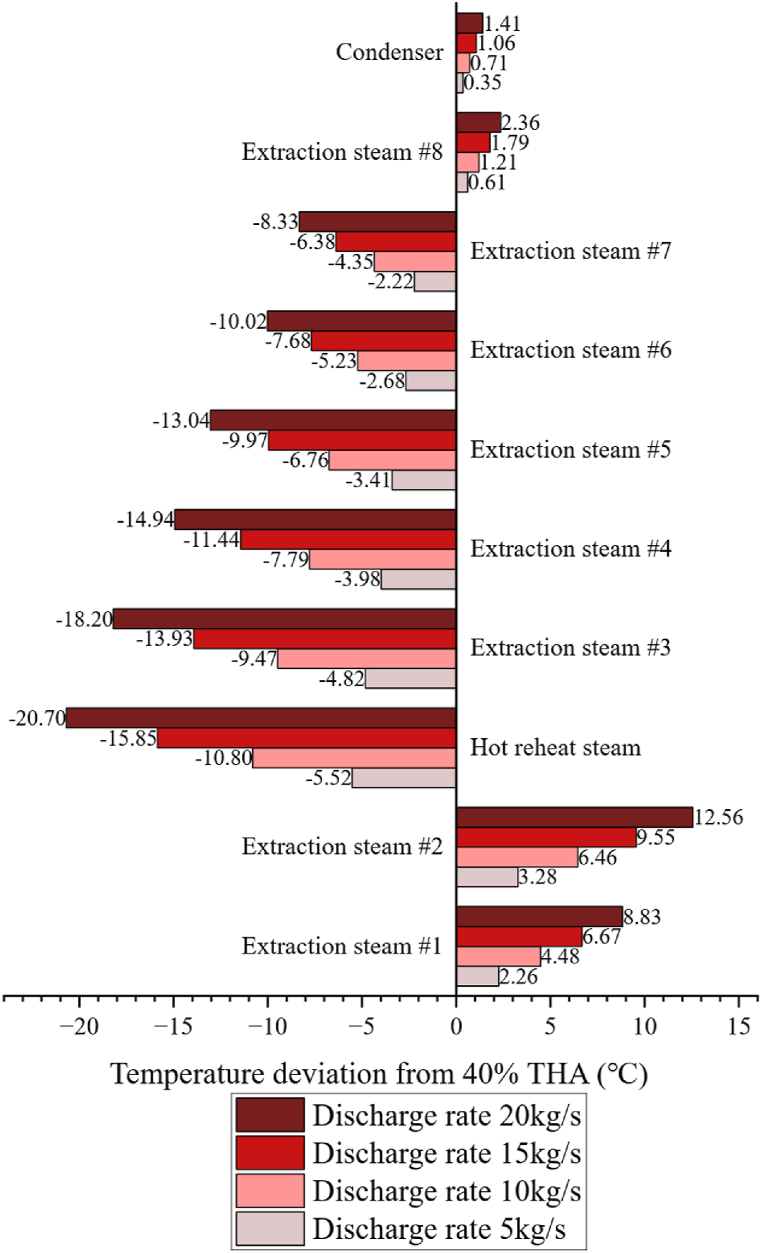
The impact of discharging to CRH on steam temperatures.

The release of saturated steam to the turbine is expected to affect the steam temperature. For the sake of argument, assume that the SA is discharging saturated steam to the turbine in accordance with the parameters set out in Plan A, from a fully charged state. Table 3 displays the distribution of steam temperature at 40 % THA. Fig. 5 shows the impact of different discharge rates on steam temperatures. When steam is released to CRH, the effect on the steam temperature in HPT and LPT is similar, while the effect on IPT steam temperature is the greatest. At the release rate of 20 kg/s, the difference between the IPT inlet temperature and the live steam temperature will be 37.6 °C. It is not feasible to have a higher release rate as this temperature difference is limited to 42 °C at 40 % THA. Therefore, the maximum discharge rate to CRH is set to be 20 kg/s. A higher discharge rate of 35 kg/s can be applied when discharging to the LP turbines, as the working condition there is relatively more moderate.

**Table 3:** Steam temperature at 40 % THA.

Location	Temperature(°C)
Live steam	599
Hot reheat steam	582.1
Extraction steam #1	406.85
Extraction steam #2	377.41
Extraction steam #3	473
Extraction steam #4	341.92
Extraction steam #5	251
Extraction steam #6	141.19
Extraction steam #7	86.046
Extraction steam #8	47.684
Condenser	32.326

To ensure that the proposed SA provides sufficient storage capacity, the discharge time of the SA is designed around 30 min. The volume of the SA *V*_B_ can be estimated by the steam mass provided by the SA during discharging *m*, the index 1 stands for the fully charged state, the index 2 stands for the fully discharged state, and *β* is the max volume fraction of liquid water. The SA volume is

(23) $$V_{B}=\frac{m\nu_{1}^{\prime }}{\beta }\frac{0.5\left(h_{1}^{\prime \prime }+h_{2}^{\prime \prime }\right)-h_{2}^{\prime }}{h_{1}^{\prime }-h_{2}^{\prime }}$$

The RTE of the proposed SA can be defined by the net power increase during discharging process and the net power decrease during discharge process:

(24) $$\begin{array}{c} RTE=\frac{\int P_{\text{set}}-P_{\text{discharging}}dt}{\int P_{\text{charging}}-P_{\text{set}}\;dt} \end{array}$$

The thermodynamic performances of the proposed schemes are shown in Table 4. The relationship between SA volume and the discharge rate release is proportional. The power increase is also linearly proportional to the discharge rate. An increase in the discharge rate results in a reduction of RTE, although the effect is small. The selection of charging and discharging nodes has the greatest impact on RTE, and Scheme B achieves a lower RTE. This is because releasing steam to the LPTs will result in a pressure rise at the IPT's outlet, causing a shaft power decrease for both LPTs and HPT. Releasing steam to the CRH, on the other hand, only reduce the shaft power of HPT. For the same reason, releasing steam to the IPT can provides a longer discharge time with a significantly smaller discharge rate, and the same time providing more power increase. In addition, using higher qualitied steam charge source led to a significantly higher recharge speed, the SA containers can be also made smaller.

**Table 4:** Thermodynamic performance of the SA.

Parameters	Scheme A1	Scheme A2	Scheme A3	Scheme A4	Scheme B
SA volume	300 m³	225 m³	155 m³	75 m³	450 m³
Max volume fraction of liquid water	0.9	0.9	0.9	0.9	0.9
Design pressure	8 MPa	8 MPa	8 MPa	8 MPa	2.5 MPa
Water refill rate	50 kg/s	50 kg/s	50 kg/s	50 kg/s	50 kg/s
Water refill time	195.44s	148.44s	102.33s	49.53s	155.88s
Steam charge rate	50 kg/s	50 kg/s	50 kg/s	50 kg/s	50 kg/s
Steam charge time	524.16s	397.56s	274.07s	132.67s	1034.82s
Steam discharge rate	20 kg/s	15 kg/s	10 kg/s	10 kg/s	35 kg/s
Steam discharge time	1800s	1820s	1883s	1823s	1702s
Average power decrease during discharging and water refilling	8.4%Pe	8.4%Pe	8.41%Pe	8.41%Pe	7.3%Pe
Average power increase during charging	2.13%Pe	1.6%Pe	1.09%Pe	0.55%Pe	2.01%Pe
Round-trip efficiency (RTE)	63.60 %	64.35 %	64.90 %	65.64 %	54.70 %

All of these factors make scheme A a preferable choice over scheme B. Therefore, only scheme A will be discussed further.

**Fig. 6:**
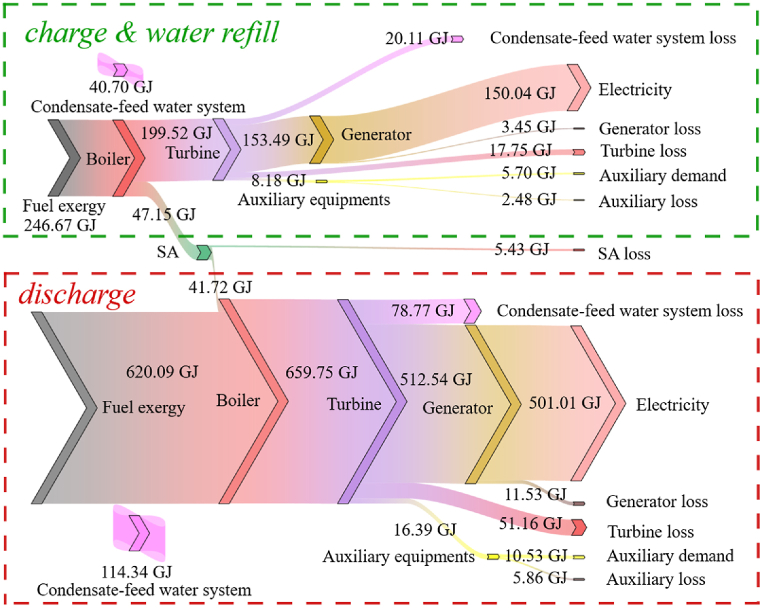
Exergy flow during a round trip.

Another important measure of energy storage devices is their exergy efficiency. The exergy flow of scheme A1 during a round trip is shown in Fig. 6. During a round trip, the SA produces 5.43 GJ of exergy loss, which is 2.52 % of the total exergy loss in the turbine side of the unit. Define the turbine exergy efficiency $\epsilon_{t\text{urbine}}$ in equation (25), where $Ex_{\text{electricity}}$ is the electricity power output, $Ex_{\text{turbine}}$ is the net exergy flow in the turbine.:

(25) $$\epsilon_{t\text{urbine}}=\frac{Ex_{\text{electricity}}}{Ex_{\text{turbine}}}$$

Compared to the fixed 40 % THA condition, the turbine exergy efficiency decreased from 76.64 % to 75.77 % during a round-trip, resulting in an exergy loss of 7.54 GJ. This indicates that the turbine system, rather than the SA, is responsible for the additional exergy loss during a round-trip.

It must be stated that the steady-state thermodynamic calculations did not consider the boiler heat transfer model and could not capture the transient characteristics of the system. Further discussion is required regarding the performance of the SA during power ramp operations.

## 4 Improvements in the power ramp rates

Prior to this study, the combustion, flue gas, water-steam and electrical modelling, as well as the control systems such as CCS and DEH of the reference CFPP were carried out using SimStore software. The model includes the boiler side water-steam system (water wall, superheat, reheat, desuperheating and bypass system), the flue gas system, the milling system, the combustion system, the turbine system, and the condensate-feedwater system. The software employs a flow net method, which enables real-time simulation. And the simulation of the DCS is based on a translation of Emerson's Ovation platform. The model is validated using data collected from the reference plant (see Table A1 - Table A3) and the results show that the simulated values are consistent with the actual operating conditions over a relatively wide unit load range.

The plant's power ramp rate under CCS control is an important indicator of its flexibility performance. Under CCS control, the turbine automatically adjusts the live steam valve opening according to the sliding pressure curve (live steam pressure set to 8 MPa when unit load is lower than 20 % THA, 10.5 MPa when unit load is 40 % THA, 25 MPa when unit load is 100%THA, while set pressure ramp rate is 0.5 MPa/min), the boiler automatically adjusts the firing rate by controlling the coal feed rate and fuel-air ratio. To increase plant flexibility, the control strategy of the SA is designed to assist the unit in responding to power ramp commands. The SA should increase the plant's power ramp rate as much as possible without breaching safety limits.

The load decrease and load increase procedures of CFPP differ significantly. Control logics should be designed for the SA to fit the needs of different power ramp procedures. Unlike discharging saturated steam to the regenerative systems as in Refs. [27,28], the operation of the bypass SA is a new concept and cannot be referenced from existing operation of extraction steam throttling. This section provides examples of various charging and discharging strategies that can enhance the unit's power ramp capability, along with their impact on power ramp rates.

### 4.1 Improvements during load decrease

The live steam pressure indicates the amount of heat energy entering the turbine. During load decrease, the steam turbine of the plant quickly lowers its steam flow rate by turning down the steam valve. The firing rate of the boiler, however, decreases much slower because of the thermal and combustion inertia. The heat energy that exceeded the limit during this transient caused an increase in live steam pressure, leading to the closure of the live steam valve and a further increase in pressure. This cycle leads to an increase in pressure, while the set value for live steam pressure is supposed to decrease. The increase in pressure also poses a risk to the plant's security. Fig. 8 shows an example of a unit's loading down process from 40%THA to 30%THA in a power ramp rate of 33 MW/min During the process, the live steam pressure quickly climbed up, triggering a pressure lock, locking the unit load command for about 43 s. The pressure lock indicated that the set power ramp rate was beyond the unit's power ramp capability.

**Fig. 7:**
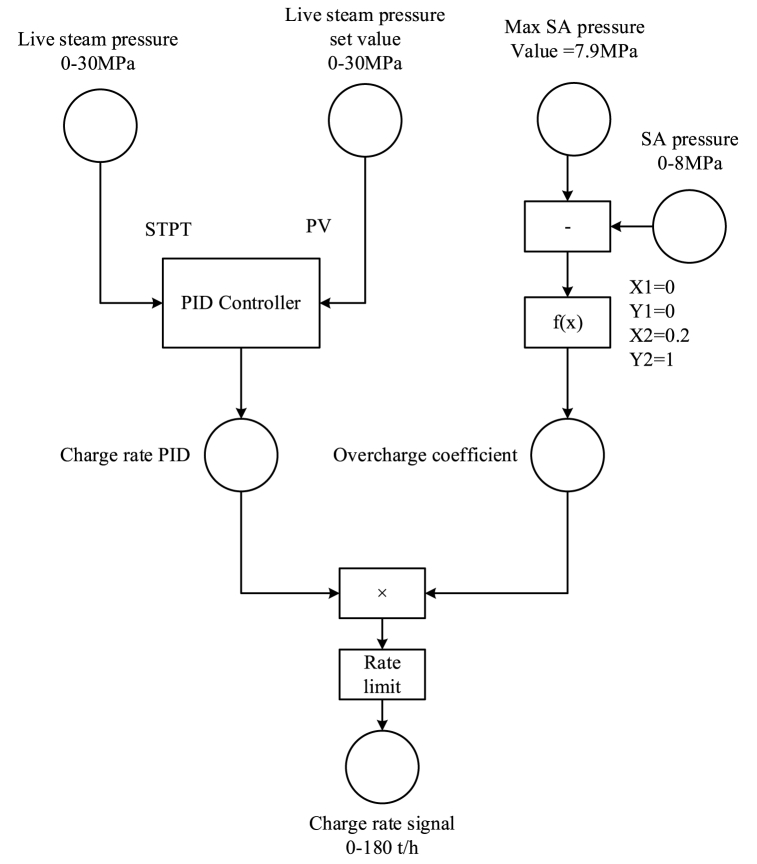
Control scheme of the SA's charge strategy while loading down.

To improve the load decrease rate, a PID controller was implemented, using the live steam pressure as the control variable, as shown in Fig. 7. To prevent overcharge, the steam flow rate is automatically reduced until it stops when the SA is about to be filled. Using steam flow rather than valve opening makes it easier to implement mass flow restrictions. It also improves the flexibility of the controller to adapt to different load conditions.

**Fig. 8:**
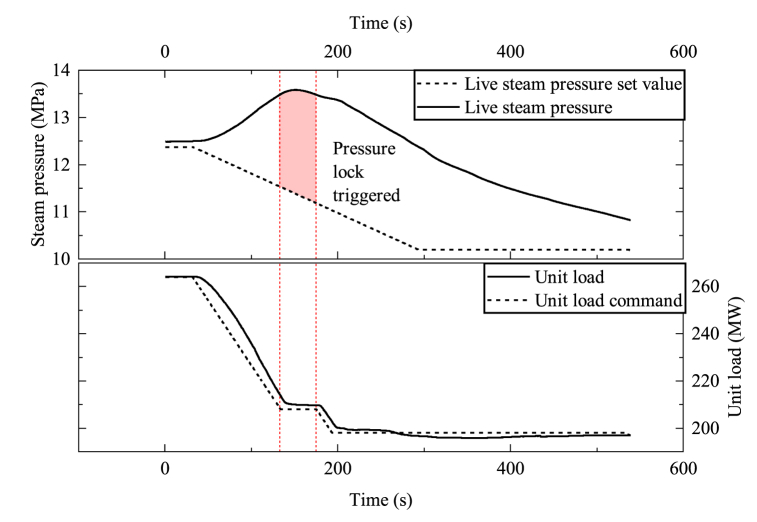
The unit's response to a load decrease command of 33 MW/min.

The SA is initially at a fully discharged condition, where the initial SA pressure is equal to the CRH and the water has already been re-filled. The unit was tested to determine its response to a load decrease command. As shown in Fig. 10, the control of both live steam pressure and unit load were improved. It took approximately 268 s for the live steam pressure to stabilize at the new set value. Without the SA, this time would have been 596 s. Additionally, the unit can now adjust load more quickly without concerns about the security of the live steam pressure, as drastic pressure rises no longer occur.

Define the accumulated live steam pressure deviation before the pressure stabilizes *I*_*p*_ and the accumulated deviation of unit load before the unit load stabilizes *I*_w_ as indicators of control quality:

(26) $$I_{p}=\int \left|P_{\text{real}}-P_{\text{set}}\right|dt$$

(27) $$\begin{array}{c} I_{w}=\int \left|W_{\text{real}}-W_{\text{set}}\right|dt \end{array}$$

**Fig. 9:**
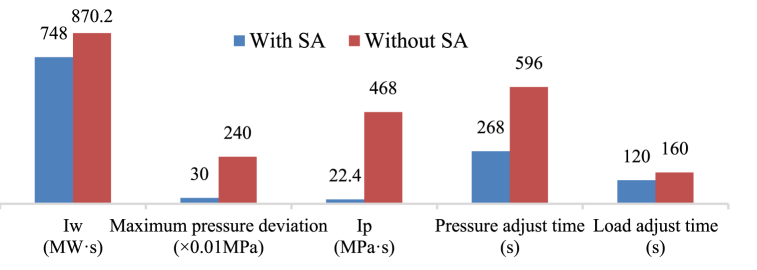
Improvements during load decrease operation.

**Fig. 10:**
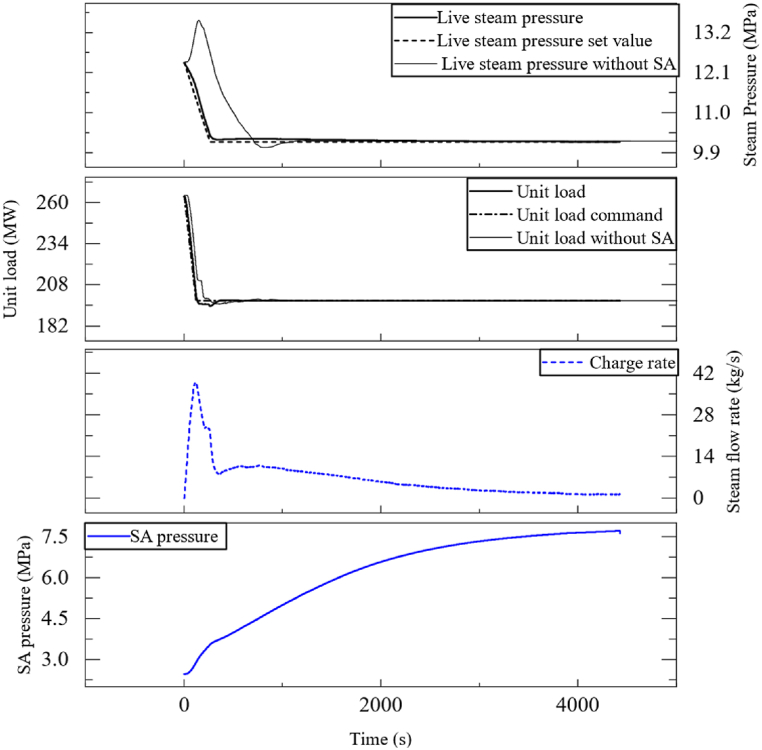
The SA's response to a load decrease command of 33 MW/min.

Improvements during load decrease procedure using a SA are shown in Fig. 9, Fig. 10.

### 4.2 Improvements during load increase

**Fig. 11:**
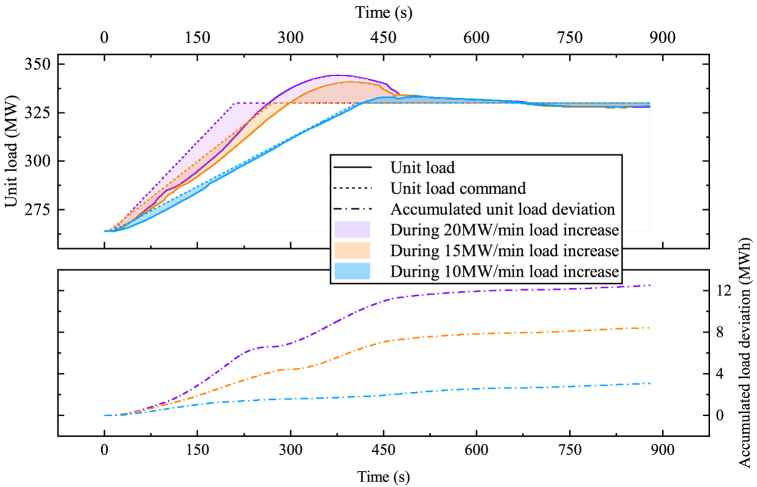
The unit's response to different load increase commands.

Due to the thermal and combustion inertia in the boiler, the unit's load increase rate is generally lower than the load decrease rate. As demonstrated in Fig. 11, the unit responded well to a load increase command of 10 MW/min from 40 % THA to 50 % THA. However, the unit is unable to follow load increase command of higher than 15 MW/min.

The distinction between the unit load and unit load command arises from inadequate heat supply. In load increase procedures, the difference is used as a control input for the PID controller. The temperature of HRH drops during discharge, a temperature coefficient is used to ensure that the deviation between live steam and HRH temperatures does not exceed the limits.

PID control typically results in some degree of overshoot. To mitigate this overshoot, a secondary PID controller is employed to regulate the steam charge flow.

**Fig. 12:**
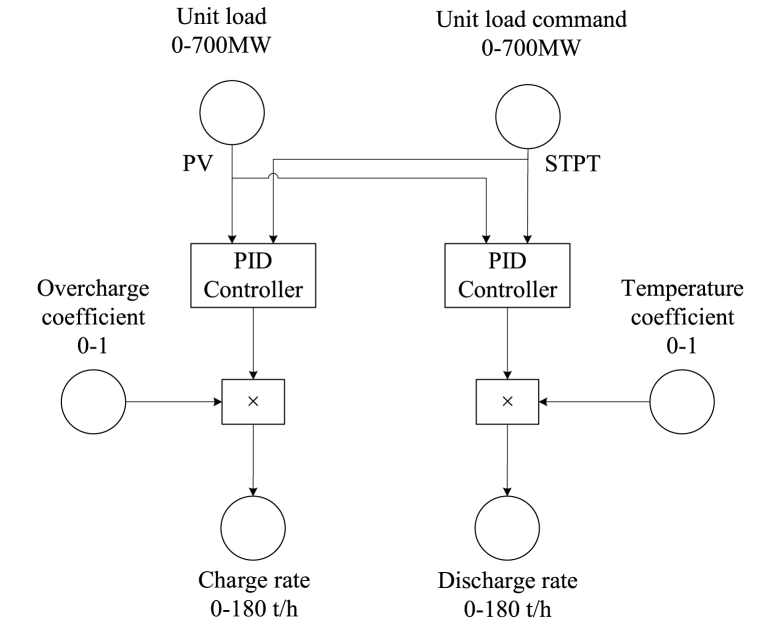
The SA's control strategy while loading up.

**Fig. A2:**
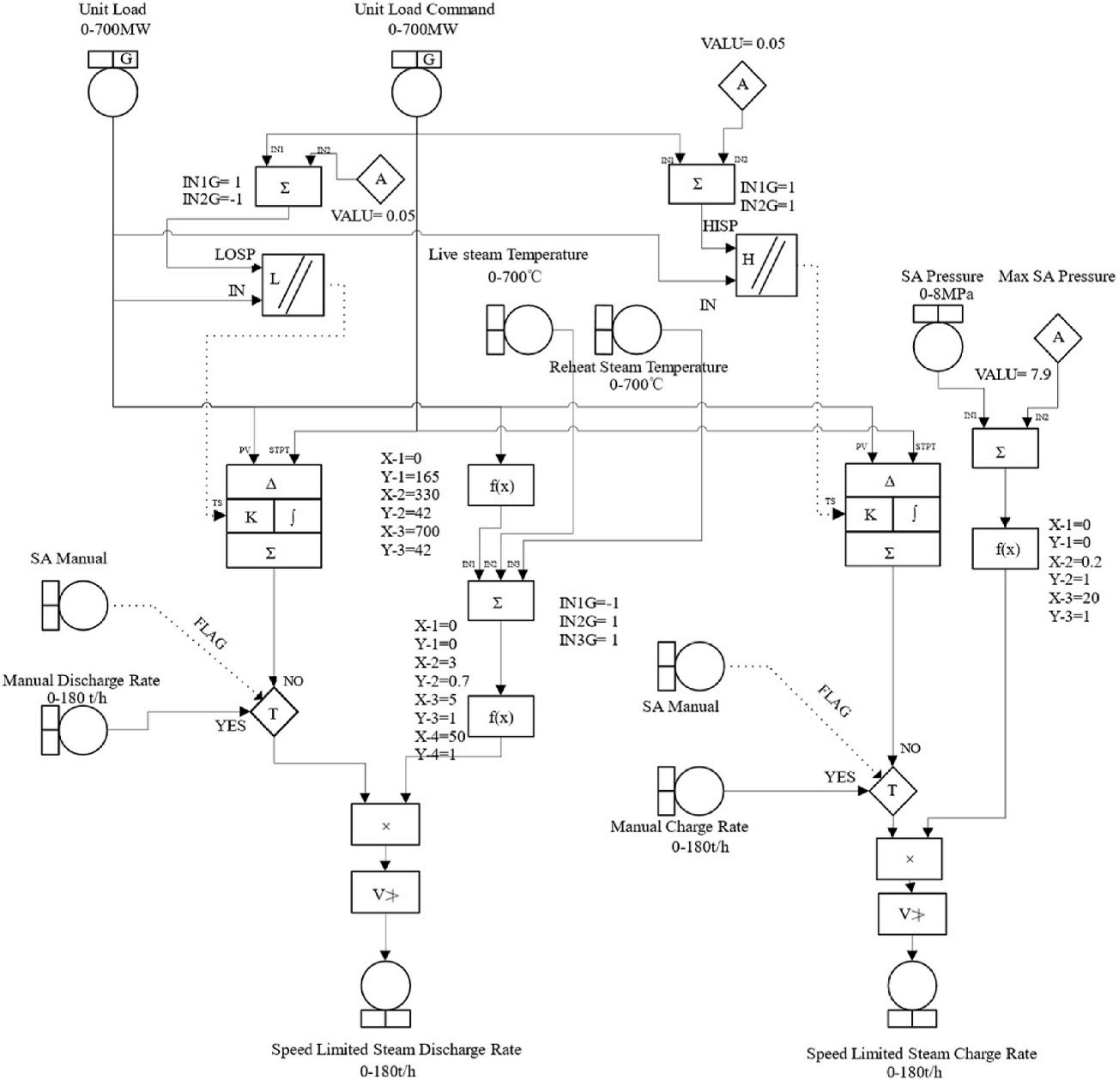
Detailed SA control logic during load increase.

The reason for using a different control input during load increase than during load decrease is that the SA release does not directly affect the live steam pressure. However, it has a direct effect on the turbine, boosting power. The control scheme is shown in Fig. 12, a more detailed scheme is presented as Fig. A2.

**Fig. A3:**
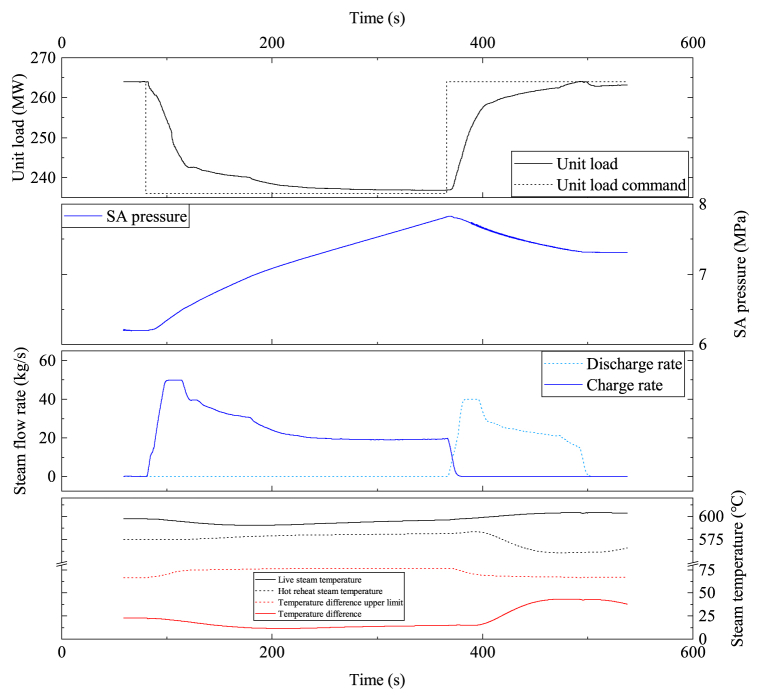
The unit's response to power ramp commands using the SA.

Similar control strategy can be adopted to provide primary frequency reserve, as shown in Fig.A3. The potential for primary frequency reserve has already been discussed in the thermodynamic performance section of this article, readers can refer to Table 4.

**Fig. 13:**
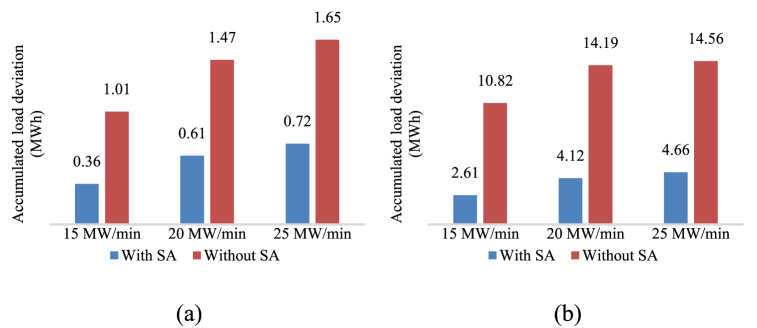
(a) Control improvements of load deviation; (b) Control improvements of load overshoot.

**Fig. 14:**
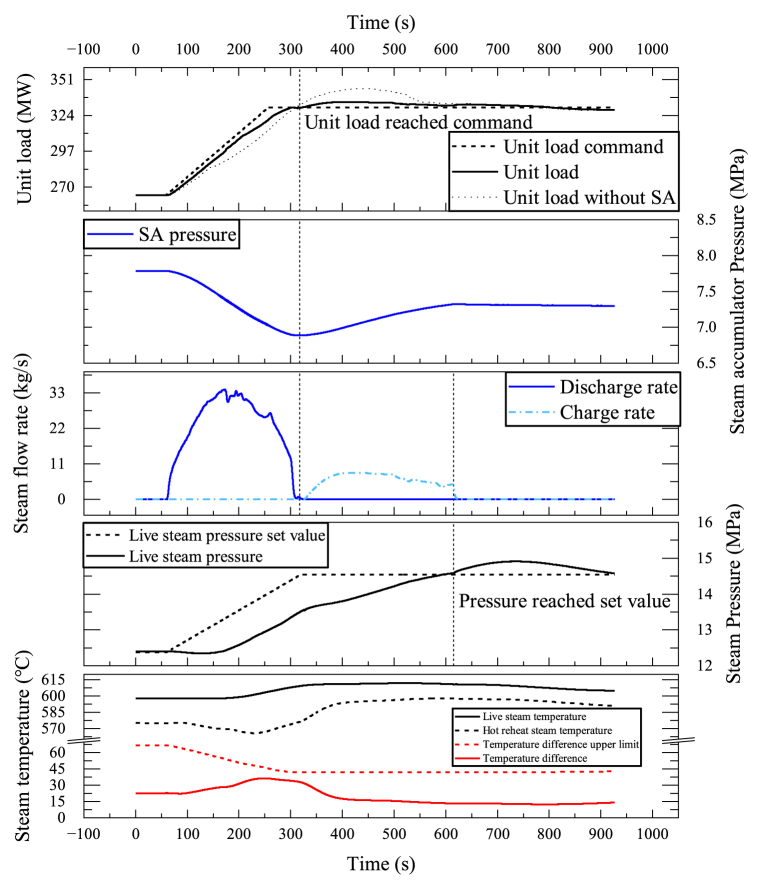
The SA's response to a load increase command of 20 MW/min.

The SA is initially at a state near fully charged to observe the SA's performance during a load increase command of 20 MW/min from 40 % THA to 50 % THA. The improvements during load increase procedures are shown in Fig. 13, Fig. 14. With the SA, the unit load can well follow the 20 MW/min power ramp command. The SA reduced both load deviation and overshoot. However, the live steam pressure shows grater deviation greater deviation because of the HPT back pressure rising during discharge. As anticipated, the temperature differential between live steam and HRH exhibited an upward trend. However, the temperature coefficient successfully constrained it within a reasonable range.

**Fig. 15:**
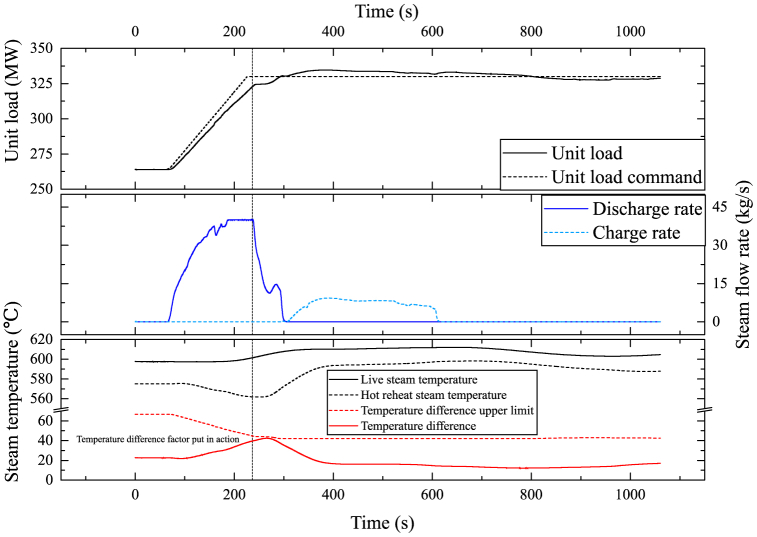
The impact on steam temperature of an exceeded load increase command of 25 MW/min.

However, the SA is unable to support a load increase rate greater than 20 MW/min. When responding to load increase command of 25 MW/min, as shown in Fig. 15. The temperature difference between live steam and HRH at 237 s exceeded upper limit. This means the SA has released all its potential, yet the unit load cannot follow the command. The temperature difference can be manually optimized to extend the SA's potential of raising the power ramp speed, nonetheless, the SA cannot provide unlimited load flexibility.

## 5 Results and discussions

Most research on deep peak-shaving of CFPPs focuses on the safety and stability of the unit operation. However, there are only a few studies on the power ramp flexibility performance. Apart from the operation regulation and control of the regenerative system, whose potentials are almost exhausted by the growing need for power ramp flexibility, the integration of external energy storage into the water-steam cycle provides an efficient approach to increase the plant flexibility.

This paper presents the successful integration of a novel steam accumulator heat storage device to the bypass system of a 660 MW supercritical CFPP, which enhances the power ramp rate at low load. A thermodynamic calculation at 40 % THA proved the feasibility of the proposed steam accumulator. Further simulations were carried to test the system performance of the proposed bypass steam accumulator during power ramping operations. The improvements in the main parameters resulting from the integration of the proposed bypass steam accumulator are presented in Table 5 and Table 6. The results show that the proposed integration scheme and its control method effectively improved the flexibility of the CFPP. The main findings are.

1) Under low load, the CFPP possess low energy storage in the water-steam cycle, and the energy flow to the regenerative system is weaken. When integrating the energy storage into the bypass system, the unit load has less influence on the discharge rate of the energy storage, thus allowing higher discharge rate. For instance, replacing the extraction steam #2 with the discharged saturated steam at the unit load of 40 % THA results in a discharge rate of 11 kg/s, while the feasible discharge rate to the cold reheat steam is 20 kg/s.
2) Discharging to the intermediate turbine result in both higher round-trip efficiency and higher flexibility improvement. According to the Stodola-Flugel equation, the extra steam flow from the steam accumulator raises the pressure level of the integration point. The compression ratio of the thermocompressor makes this pressure rise even higher. Discharging steam to the low pressure turbine causes efficiency reduce in both high pressure turbine and intermediate pressure turbine, thus is less efficient.
3) Achieving higher load decrease rate is mainly limited by live steam pressure control. Integrating the steam accumulator into the bypass system enables direct control on live steam pressure. The proposed control method provides a simple yet effective routine to improve the load decrease rate.
4) Load increase rate is limited by the inertia of the combustion and heat transfer process. Additional energy source should be applied to support the energy balance. Steam accumulator provides fast response in a range of seconds, thus results in instant load response. The proposed control method assists the load increase operations by responding to the load deviation.
5) The reduction in reheat steam temperature during the discharge period is the limiting factor in further increasing the unit load increase rate, because the temperature difference between live steam and the reheat steam causes heat stress at the turbine inlet. Note that the initial live steam - hot reheat steam temperature difference of the unit in this case is 22.7 °C, which is a high value. It is possible to regulate the temperature difference in actual operation, and the unit's loading rate can be further increased.

**Table 5:** Thermodynamic characteristic of the proposed steam accumulator.

Parameters	
Charging steam source	Live steam pipe
Discharge outlet	Cold reheat steam pipe
Water source	Feed water pump outlet
Volume	300 m³
Design pressure	8 MPa
Storage time at maximum discharge rate	30 min
Maximum discharge mass flow rate	20 kg/s
Average power decrease during discharging and water refilling	8.4%Pe
Average power increase during charging	2.13%Pe
Round-trip efficiency (RTE)	63.60 %

**Table 6:** Main results on power ramp transients.

Parameters	Original	With Steam accumulator
Maximum load decrease rate at 40 % THA	4 % Pe/min	>5 % Pe/min
Maximum live steam pressure deviation during load decrease	2.4 MPa	0.3 MPa
Maximum load increase rate at 40 % THA	1.5 % Pe/min	3 % Pe/min
Maximum load overshoot during 3 % Pe/min load increase	14.19 MW	4.12 MW
Maximum live steam – hot reheat steam temperature difference during 3 % Pe/min load increase	22.71 °C	36.39 °C

The evaluations are based on a typical 660 MW supercritical CFPP, the results are mostly transferable to units built during year 2005 to year 2015 with similar boiler parameters and are qualitatively referrable to other CFPPs. Further research includes control optimization and switching of the SA within the full load segment, the bypass SA's impact on the frequency control of the unit, and the bypass SA's utilization on combined heat power (CHP) units.

## Data availability statement

The authors do not have permission to share data.

## CRediT authorship contribution statement

**Hongyu Ding:** Writing – original draft, Validation, Software, Methodology. **Sibian Ding:** Validation, Software, Data curation. **Qingxiong Tan:** Validation, Software. **Cheng Zhang:** Conceptualization. **Qingyan Fang:** Supervision, Conceptualization. **Tao Yang:** Writing – review & editing, Project administration, Investigation, Funding acquisition, Conceptualization.

## Declaration of competing interest

The authors declare that they have no known competing financial interests or personal relationships that could have appeared to influence the work reported in this paper.

## Figures and Tables

**Fig. A1:**
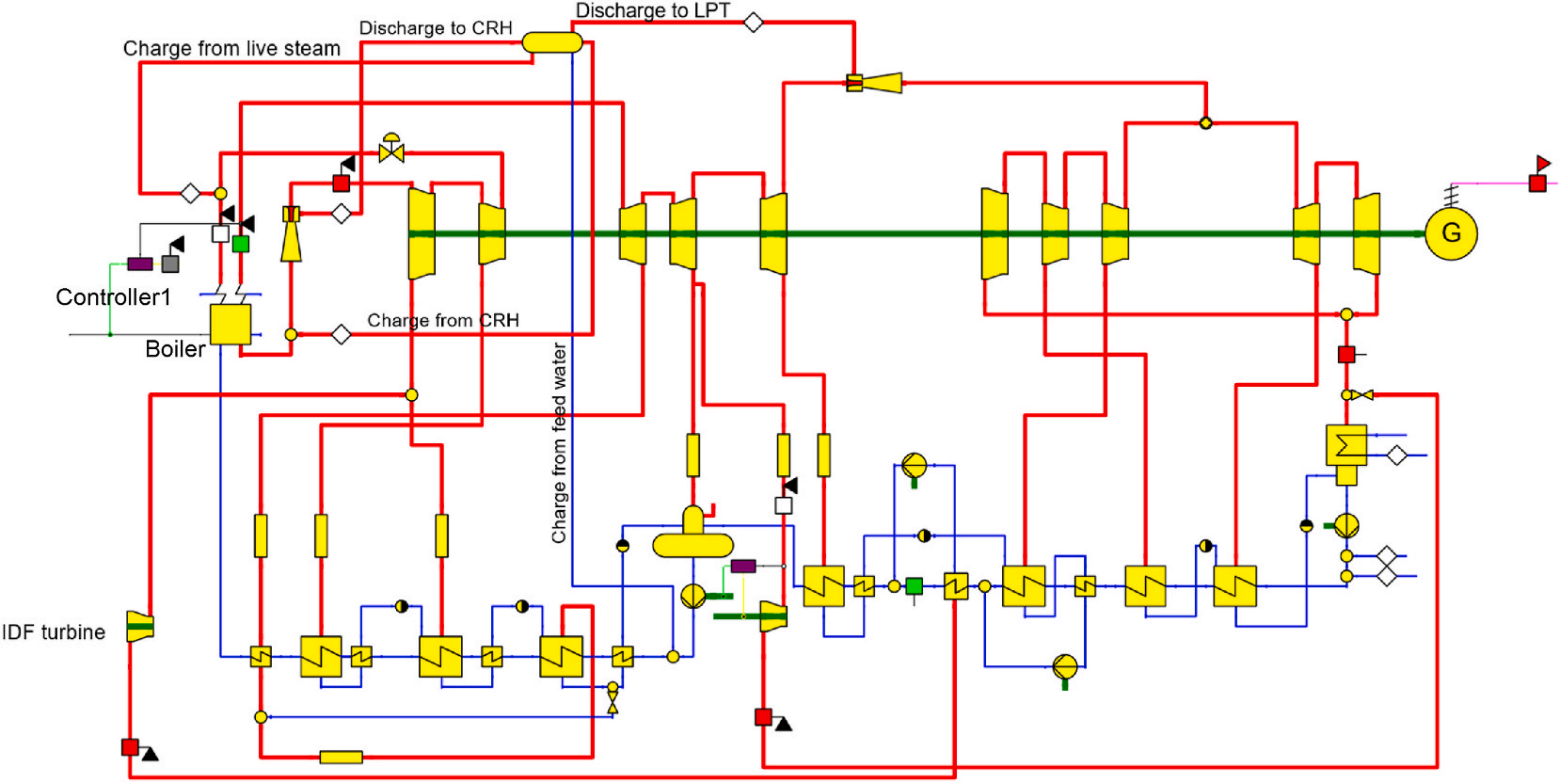
Thermodynamic model.

**Table A1:** SimStore model accuracy at 100 % THA condition

Parameter	Measured	Model	Error (%)
Unit load (MW)	662	662.4	0.06
Feed water rate (t/h)	1859	1902	2.31
Live steam flow rate (t/h)	1862.1	1890.01	1.50
Live steam pressure (MPa)	25	25.85	3.40
Live steam temperature (°C)	603.6	605.3	0.28
Hot reheat steam pressure (MPa)	4.79	4.77	−0.42
Hot reheat steam temperature (°C)	603.5	605.2	0.28

**Table A2:** SimStore model accuracy at 75 % THA condition

Parameter	Measured	Model	Error (%)
Unit load (MW)	403.5	404.5	0.25
Feed water rate (t/h)	1398	1438	2.86
Live steam flow rate (t/h)	1436.9	1419.62	−1.20
Live steam pressure (MPa)	19.65	20.87	6.21
Live steam temperature (°C)	591.8	599.2	1.25
Hot reheat steam pressure (MPa)	3.88	3.71	−4.38
Hot reheat steam temperature (°C)	597.9	604.4	1.09

**Table A3:** SimStore model accuracy at 50 % THA condition

Parameter	Measured	Model	Error (%)
Unit load (MW)	403.5	404.5	0.25
Feed water rate (t/h)	1398	1438	2.86
Live steam flow rate (t/h)	1436.9	1419.62	−1.20
Live steam pressure (MPa)	19.65	20.87	6.21
Live steam temperature (°C)	591.8	599.2	1.25
Hot reheat steam pressure (MPa)	3.88	3.71	−4.38
Hot reheat steam temperature (°C)	597.9	604.4	1.09

## Appendix 1

Fig.A1 shows the thermodynamic model built using Ebsilon software. It is similar to Fig. 1, but with more details on boundary conditions and assumptions. A controller is applied to the boiler. It ensures the water-steam cycle obtain the same amount of heat as 40 % THA condition, according to the third assumption in section 3.

Table A1-Table A3 compares some of the key parameters at certain loads. The parameters marked as “measured” are acquired from the reference plant, and the parameters marked as “model” are simulation results of the SimStore model.

Fig.A2 shows the detailed control logic during load increase. The unit load generates the current upper limit of the temperature difference between live steam and reheat steam, which is defined by the operation regulation.

Fig.A3 shows the unit's response to power ramp commands using the SA. This process uses a control logic similar to that during load increase.
